# Health Technology Assessment: informed by science or in the service of politics?

**DOI:** 10.11606/s1518-8787.2021055003234

**Published:** 2021-10-13

**Authors:** Patrícia Coelho de Soárez

**Affiliations:** I Universidade São Paulo Faculdade de Medicina Departamento de Medicina Preventiva São PauloSP Brasil Universidade São Paulo. Faculdade de Medicina. Departamento de Medicina Preventiva. São Paulo, SP, Brasil

**Keywords:** Biomedical Technology Assessment, Technology Assessment, Science, Technology, and Health Innovation Management, National Science, Technology, and Innovation Policy, Science, Technology, and Society

## Abstract

The youth of Health Technology Assessment (HTA), as an institutional policy at the national level, signals the need to reflect on how its implementation took place under the perspective of its insertion in health policy and the scientific field. At the end of its first decade, these questions arise: has HTA translated into a health policy informed by science? Has its scientific foundation been used in the service of politics? To understand this political process, we apply the multiple-streams framework formulated by John Kingdon. The use of science to inform policy and the political use of science present themselves in an unstable balance. The survival of this policy will depend not only on science but on the art of orchestrating the interests of various agents so that HTA becomes a health policy for strengthening and sustainability of SUS.

## INTRODUCTION

The National Commission for Technology Incorporation in the Unified Health System (CONITEC) has become the centerpiece of the field of Health Technology Assessment (HTA) in Brazil. Its normative acts and official speech reiterate the importance of preparing technical reports based on scientific evidence, economic evaluation, and analysis of the impact of technologies adoption and the analysis of documents by deliberative processes. All instances of the Unified Health System (SUS) must share these processes. Also, they must be widely disseminated in society, allowing social participation in the decision-making process for technologies adoption into the SUS^1^.

As an institutional policy at the national level, the HTA youth signals the need for a reflection on how its implementation occurred: from the perspective of its insertion in the health policy in general, and conversely, under the scientific field, which produced the essential science and technique for this institutionalization. At the end of its first decade, these questions arise: has HTA translated into a health policy^a^ informed by science? Was its scientific foundation used in the service of politics^b^?

### HTA Policy Formulation and Implementation Process

To understand this political process, the multiple-streams framework formulated by John Kingdon proves convenient. This theory argues that the entry of a new problem on the government’s agenda requires the confluence of three independent streams that cross the decision structures: the problem stream, the policy stream, and the political stream^2^. The problem stream and the policy stream contain, respectively, issues that require public attention and ideas and technical proposals for solving the problems. The politics stream comprises political transitions and social pressure. When the three streams come together, windows of opportunity emerge, and governments decide to act^3^.

In the case of HTA, the judicialization of public health was recognized as a fact whose problematic repercussions strengthened arguments in favor of implementing a policy that sought to resolve it. In 2005, the “judicialization epidemic” began: many lawsuits against the Brazilian Ministry of Health (MS), whose climax culminated in a public hearing on the judicialization of health within the SUS, convened by the President of the Supreme Court in 2009^4^.

Since the 1990s, HTA was already a topic discussed by MS technical staff, but its relevance was not sufficiently recognized to be translated into actions and practices. In 2003, the administrative reform carried out in the MS suggested attention to strategic inputs, scientific and technological development, with the institution of the Secretariat of Science, Technology, and Strategic Inputs (SCTIE)^5^.

In the following year, society groups interested in the topic began to design possible alternatives, starting the policy stream. During the 2^nd^ National Conference on Science, Technology, and Innovation in Health, the HTA was recognized as a strategic instrument to support technology management in health. For the first time, a specific policy in this field is proposed, resulting in the constitution of the Commission for Proposal Preparation for the National Policy on Health Technology Management (PNGTS) in 2005. In 2006, the MS created the Commission on Technology Incorporation (CITEC), the first body responsible for managing the process of incorporating technologies and supporting decision processes in SUS^6^. The standards suggested by Kingdon were present: technical reliability, acceptability, and compatibility with current values in society at that time.

As for the politics stream, that was a favorable moment for the implementation of public policies, since in 2007, both the Secretary of Science, Technology and Strategic Inputs, as the Minister of Health, supported the idea of a policy based on scientific rationality to guide the technologies adoption into the SUS. The policy formulation process was able to advance as the values and interests that guided the policy, and the political values and interests coincided. It culminated in the publication of the PNGTS in 2010^7^.

Then followed a political move to structure a body responsible for adopting health technologies into the SUS. The unlikely combination of two almost antagonistic bills, 338/2007 and 219/2007, gave rise to Federal Law 12401/2011, which created CONITEC and determined that the SUS would only provide treatments included into public policies^8^.

The integration of the three streams (problem, policy, and politics) opened the window of opportunity for the creation of CONITEC, which instituted the formal role of the HTA in the decision-making processes of the SUS. CONITEC managed to implement in a short period the steps of the HTA process – the adoption stream, the rituals in the expanded composition plenary, the deadlines for analysis and recommendation, the public consultation, and the availability of recommendation reports. From January 2012 to September 2020, of the 717 requests for adoption of technologies received, 558 (78%) received a final recommendation from CONITEC^9^.

Concerning the scientific field, CONITEC’s recommendations are under critical analyses within the scope of scientific and technical quality in light of what the law dictates. The arguments focus on evaluating the quality and consistency of the recommendation reports and the transparency of the plenary’s deliberative process, including investigating the actors involved and the basis for their choices. The lack of publicity by the institutions responsible for preparing the reports has also been the target of criticism^10,11^.

From the perspective of political agents directly interested in adopting particular technologies, criticisms on the decisions are frequent, moreover, on what is considered a delay in adopting technologies presented as essential for patients with specific diseases. Thus, a questioning arises concerning the legitimacy of the policy of HTA as a whole.

Two main risks may have occurred in CONITEC’s HTA process: the risk of bias and the risk of incompleteness. The bias risk materializes when a distorted image of the evaluated technologies’ properties or value appears due to gaps in the available evidence or due to evidence interpretation or synthesis^12^. The process of incorporating Nusinersena, the most expensive drug product ever incorporated into the SUS, exemplifies this risk of bias. A flexibilization of the rite not supported by the available scientific evidence was established^11^.

As for the risk of incompleteness, specific technology evaluation criteria are neglected or not sufficiently recognized. This was the case of the recommendation to adopt six medicines into the SUS without registration with the Brazilian Health Regulatory Agency (ANVISA), i.e., without an assessment of their safety and efficacy^13^. Moreover, the risk of incompleteness in the incipient use of economic evaluations in recommendation reports was present. In these cases, eighty-eight (87.1%) technologies received a favorable recommendation for adoption without a full economic evaluation with the incremental cost-effectiveness ratio estimate^10^.

### Development of the Scientific Field in Brazil

The institutionalization of the “evidence-based/informed” policy was possible due to the training of human resources and the formation of research networks strongly stimulated and financed by the Ministry of Health. The country formed a broad group of experts on the basic concepts and methods used in HTA studies^14^ and also an epistemic community^c^, or a scientific field, in a sense given by Bordieu^15,16^. There are dominant actors responsible for the rules of the field, for the development of national methodological guidelines, and for conducting the studies that instrumentalised CITEC and CONITEC. They dispute the legitimacy to participate in policies implementation and research funding, strongly directed by the MS.

Some of the seminal epistemic actors were academics and participated in government acting as critical catalysts for HTA. The influence of this initial group of specialists on decision-makers diminished with the expansion and diversification of new areas added to the field. The internal dynamics of this group reveals a fragile cohesion, a product of the diversity of fields of origin or activity (Public Health, clinical area, pharmacy, economics) of these professionals.

Human resources training is necessary to face the current challenges of evaluating technological innovations, for example, for rare diseases^17,18^, and to deal with future challenges related to the expansion of precision medicine (PM) in the next decade^19^. An enlarged group of researchers trained in traditional modeling approaches, such as Markov models, has not yet been consolidated. The challenge of moving towards modeling with dynamic simulation capable of capturing the individual care trajectories of PM technologies is already pressing. New methods of economic evaluation of PM will be needed to deal with this complexity of cascading decisions reflected in the multiple treatment trajectories to make these methods result in significant information to support decisions in health policies^19^.

The high cost of PM technologies can exacerbate health inequalities and jeopardize the sustainability of their systems, especially in low- and middle-income countries. The evaluation of these technological innovations will require new technical knowledge and possibly another process in the HTA bodies, given the pace of innovation and the regularity of updating algorithms and applications^20^.

### Perspectives on the Past and the Present to Reflect the Future of HTA

The formulation of the PNGTS took place between 2004 and 2010, a moment of expansion of social protection and affirmative action policies. The creation of CONITEC in 2011 crystallized the institutionalization of HTA in the SUS. HTA implementation took place between 2013 and 2018, a turbulent period of democratic regression, continuous institutional degradation, and economic crisis, especially marked by 2016, the year of the democratic rupture related to President Dilma Rousseff’s impeachment and the extinction of all secretariats and ministries linked to the expansion of rights or distributive policies^21^.

The establishment of a national HTA body, such as CONITEC, is a difficult task that requires many resources and a period of maturity and political commitment to become effective and sustainable^22^. Since 2016, CONITEC has lost its political strength and faced destabilization strategies, with essential replacements in its technical staff. Also, the veiled skepticism about policies based on scientific evidence became an explicit scientific denial after the 2018 election^23^. A clear example of this position was the current government’s decision to zero the import tax rate for Zolgensma, the most expensive drug product globally – whose dose costs R$ 12 million – in July 2020, when the drug product was yet unregistered with ANVISA.

Answering the initial questions, we can say that there is a precarious balance between the use of science to inform policy and the political use of science. HTA policy has sought to be informed by science, but technical-scientific gaps must be addressed to make this process based on scientific evidence. It has been used in the service of politics and is currently at risk for not being valued in current political discourse.

The survival of this policy will depend not only on science, i.e., on the strengthening of scientific and technical quality, but above all on the art of orchestrating the interests of the political and judicial systems, and the epistemic community and civil society, to favor insertion of HTA in a health policy that strengthens the SUS.

Therefore, we expect the reversal of this pendular movement of counter-democracy and advocate that the new HTA ceases to be a government policy and establishes itself as a health and social policy capable of guiding the increasingly complex SUS decision-making processes. We hope that HTA can offer a transparent, legitimate, and fair process, contributing to access to technological innovations following the population’s needs and the social value of these technologies, in compliance with the principles of universality, equity, and integrality. Thus, SUS safety, quality, and efficiency standards can be guaranteed, and its sustainability assured.

## Footnotes

a Refers to concrete material results, the configuration of policy guidelines, plans, and programs.

b Refers to the generally conflicting political negotiation processes related to the exercise of power.

c Concept operationalized by Peter Haas and Emmanuel Adler, which refers to a “network of professionals with recognized expertise and competence in a specific domain and a legitimate and policy-relevant knowledge within a domain or problem area.” The concept goes beyond the scientific community; it also includes professionals who apply the same methodology and share the same faith or belief in the truth and the applicability of particular forms of knowledge or specific truths. It resembles Kuhn’s broader sociological definition of paradigm, which is “a constellation of beliefs, values, techniques shared by members of a given community.”
