# Supplementary material for: Health Technology Assessment: informed by science or in the service of politics?
Source: Rev Saude Publica. 2021 Oct 13;55:64. doi: 10.11606/s1518-8787.2021055003234 (PMC8522711; doi:10.11606/s1518-8787.2021055003234)
Supplement: Translation

# Translation: Avaliação de Tecnologias em Saúde: informada pela ciência ou a serviço da política?

**Authors:** Patrícia Coelho de Soárez

**Affiliations:** I Brasil Universidade São Paulo. Faculdade de Medicina. Departamento de Medicina Preventiva. São Paulo, SP, Brasil

**Keywords:** Avaliação da Tecnologia Biomédica, Avaliação de Tecnologias em Saúde, Gestão de Ciência, Tecnologia e Inovação em Saúde, Política Nacional de Ciência, Tecnologia e Inovação, Ciência, Tecnologia e Sociedade

## Abstract

A juventude da Avaliação de Tecnologias em Saúde (ATS), enquanto política institucional no âmbito nacional, sinaliza a necessidade de uma reflexão sobre como se deu sua implementação, sob as perspectivas de sua inserção na política de saúde e do campo científico. Ao final de sua primeira década, levantam-se as perguntas: a ATS se traduziu em uma política de saúde (*policy*) informada pela ciência? Sua fundamentação científica foi usada a serviço da política (*politics*)? Para compreender esse processo político, aplicamos a teoria de múltiplos fluxos formulada por John Kingdon. Estabeleceu-se um equilíbrio instável entre o uso da ciência para informar a política e o uso político da ciência. A sobrevivência dessa política dependerá não só da ciência, mas da arte de orquestrar os interesses dos vários agentes, de forma que a ATS se torne uma política de saúde de fortalecimento e sustentabilidade do SUS.

## INTRODUÇÃO

A Comissão Nacional de Incorporação de Tecnologias no Sistema Único de Saúde (Conitec) tornou-se peça central do campo da Avaliação de Tecnologias em Saúde (ATS) no Brasil. Seus atos normativos e discurso oficial reiteram a importância tanto da elaboração de relatórios técnicos – embasados em evidências científicas, avaliação econômica e análise do impacto da incorporação de tecnologias –, quanto dos processos de análise dos documentos deliberativos, que devem ser compartilhados com todas as instâncias do Sistema Único de Saúde (SUS) e amplamente divulgados na sociedade, permitindo a participação social no processo de tomada de decisão de incorporação de tecnologias ao SUS^1^.

A juventude da ATS, enquanto política institucional no âmbito nacional, sinaliza a necessidade de uma reflexão sobre como ocorreu sua implementação, de um lado, sob a perspectiva da sua inserção na política de saúde em geral, e de outro, sob a do campo científico que produziu a ciência e a técnica imprescindíveis para essa institucionalização. Ao final de sua primeira década, levantam-se as perguntas: a ATS se traduziu em uma política de saúde (*policy*^a^) informada pela ciência? Sua fundamentação científica foi usada a serviço da política (*politics*^b^)?

### Processo de formulação e implementação da política de ATS

Para compreender esse processo político, a teoria de múltiplos fluxos formulada por John Kingdon se mostra conveniente. Essa teoria argumenta que a entrada de um novo problema na agenda de governo exige a confluência de três fluxos independentes que atravessam as estruturas de decisão: o fluxo de problemas (*problem stream*), o fluxo de soluções (*policy stream*) e o fluxo político (*politics stream*)^2^. O fluxo de problemas contém questões que requerem atenção pública. O fluxo de soluções contém ideias e propostas técnicas para resolver os problemas. O fluxo político é constituído por transições políticas e pressão social. Quando os três fluxos confluem, surgem janelas de oportunidade e os governos decidem agir^3^.

No caso da ATS, a judicialização da saúde pública foi reconhecida como um fato cujas repercussões problemáticas fortaleceram argumentos a favor da implementação de uma política que buscasse resolvê-la. Em 2005 começou a “epidemia de judicialização”, um conjunto avolumado de demandas judiciais contra o Ministério da Saúde (MS) cujo clímax culminou na realização de uma audiência pública sobre judicialização da saúde no âmbito do SUS, convocada pelo Presidente do Supremo Tribunal Federal em 2009^4^.

Desde os anos 1990, a ATS já era um tema discutido pelo quadro técnico do MS, mas sua relevância não era reconhecida de forma suficiente para que se traduzisse em ações e práticas. Apenas em 2003 a reforma administrativa conduzida no MS sugeriu uma atenção aos insumos estratégicos e ao desenvolvimento científico e tecnológico, com a instituição da Secretaria de Ciência e Tecnologia e Insumos Estratégicos (SCTIE)^5^.

No ano seguinte, grupos da sociedade interessados no tema começam a desenhar as possíveis alternativas, iniciando o fluxo de soluções. Durante a 2ª Conferência Nacional de Ciência, Tecnologia e Inovação em Saúde, a ATS foi reconhecida como um instrumento estratégico para subsidiar a gestão de tecnologia em saúde, e pela primeira vez é proposta uma política específica nesse campo, que resultou na constituição a Comissão para Elaboração de Proposta para a Política Nacional de Gestão de Tecnologias em Saúde (PNGTS) em 2005. Em 2006 foi criada a Comissão de Incorporação de Tecnologias do MS (CITEC), primeiro órgão responsável por gerenciar o processo de incorporação de tecnologias e apoiar processos de decisão no SUS^6^. Estavam presentes os padrões sugeridos por Kingdon: confiabilidade técnica, aceitabilidade e compatibilidade com os valores vigentes na sociedade daquela época.

Quanto ao fluxo político, vivia-se um momento favorável à implementação de políticas públicas, já que em 2007 tanto o Secretário de Ciência Tecnologia e Insumos Estratégicos, quanto o Ministro da Saúde, apoiavam a ideia de uma política baseada na racionalidade científica para orientar a incorporação de tecnologias ao SUS. O processo de formulação da política pôde avançar porque os valores e interesses que orientavam a *policy* e os valores e interesses políticos coincidiam, o que culminou na publicação da PNGTS em 2010^7^.

Seguiu-se uma movimentação política para estruturar um órgão responsável pela incorporação de tecnologias em saúde ao SUS. A combinação improvável de dois projetos de lei quase antagônicos, os projetos nº 338/2007 e nº 219/2007, originou a Lei Federal nº 12.401/2011, que criou a Conitec e determinou que o SUS forneceria apenas tratamentos incorporados nas políticas públicas^8^.

A integração dos três fluxos (problema, soluções e político) abriu a janela de oportunidade para a criação da Conitec, que instituiu o papel formal da ATS nos processos decisórios do SUS. A Conitec conseguiu implantar em um curto período as etapas do processo de ATS – o fluxo de incorporação, os rituais na plenária de composição ampliada, os prazos para análise e recomendação, a consulta pública e a disponibilização dos relatórios de recomendação. De janeiro de 2012 a setembro de 2020, das 717 solicitações de incorporação de tecnologias recebidas, 558 (78%) tiveram recomendação final da Conitec^9^.

Quanto ao campo científico, as recomendações produzidas pela Conitec têm sido analisadas criticamente no âmbito da qualidade científica e técnica, à luz do que está previsto na lei. Os argumentos se concentram na avaliação da qualidade e da consistência dos relatórios de recomendação, bem como da transparência do processo deliberativo da plenária, incluindo a averiguação dos atores envolvidos e a fundamentação de suas escolhas. A falta de publicização das instituições responsáveis pela elaboração dos relatórios também tem sido alvo de críticas^10,11^.

Na perspectiva dos agentes políticos diretamente interessados na incorporação de determinadas tecnologias são frequentes as críticas às decisões em si e no que é considerado como atraso na incorporação de tecnologias apresentadas como essenciais para portadores de doenças específicas, ou seja, há um questionamento da legitimidade da política de ATS como um todo.

Dois riscos principais podem ter ocorrido no processo de ATS da Conitec: o risco de viés e o risco de incompletude. O risco de viés é concretizado quando da apresentação de uma imagem distorcida das propriedades ou do valor das tecnologias avaliadas, devido a falhas na evidência disponível ou na maneira como a evidência foi interpretada ou sintetizada^12^. O processo de incorporação do Nusinersena, medicamento mais caro já incorporado ao SUS, exemplifica esse risco de viés. Configurou-se uma flexibilização do rito não sustentada pela evidência científica disponível^11^.

Já no risco de incompletude, certos critérios de avaliação da tecnologia são negligenciados ou não suficientemente reconhecidos. Foi o caso da recomendação de incorporação ao SUS de seis medicamentos sem registro na Agência Nacional de Vigilância Sanitária (ANVISA), ou seja, sem avaliação de sua segurança e eficácia^13^. Também houve risco de incompletude no uso incipiente das avaliações econômicas nos relatórios de recomendação. Nesses casos, oitenta e oito (87,1%) tecnologias receberam recomendação favorável de incorporação sem avaliação econômica completa com cálculo de razão de custo-efetividade incremental^10^.

### Desenvolvimento do campo científico no Brasil

A institucionalização da política “baseada/informada pela evidência” foi possível em razão da capacitação de recursos humanos e da formação de redes de pesquisa fortemente estimuladas e financiadas pelo MS. Formou-se no país um grupo amplo de conhecedores dos conceitos básicos e dos métodos utilizados nos estudos de ATS^14^ e constituiu-se uma comunidade epistêmica^c^ ou um campo científico, no sentido dado por Bordieu^15, 16^. Há atores dominantes responsáveis pelas regras do campo, pelo desenvolvimento das diretrizes metodológicas nacionais e pela condução dos estudos que instrumentalizaram a CITEC e a Conitec. Disputam pela legitimidade para participar da implementação das políticas e pelos financiamentos de pesquisas, fortemente direcionados pelo MS.

Alguns dos atores epistêmicos seminais eram acadêmicos e participavam do governo agindo como catalisadores fundamentais da implementação da ATS. A influência desse grupo inicial de especialistas sobre os tomadores de decisão diminuiu com a expansão e diversificação de novas áreas agregadas ao campo. A dinâmica interna desse grupo revela uma coesão frágil, produto da diversidade de campos de origem ou atuação (Saúde Coletiva, área clínica, farmácia, economia) desses profissionais.

Se faz necessária a capacitação de recursos humanos para enfrentar os desafios atuais de avaliações de inovações tecnológicas, por exemplo, para doenças raras^17, 18^ e para lidar com os desafios futuros relacionados à expansão da medicina de precisão (MP) na próxima década^19^. Ainda não se consolidou um grupo ampliado de pesquisadores capacitados em abordagens tradicionais de modelagem, como os modelos de Markov, e já é premente o desafio de avançar para uma modelagem com simulação dinâmica capaz de capturar as trajetórias de cuidados individuais das tecnologias de MP. Serão necessários novos métodos de avaliação econômica da MP para lidar com essa complexidade de decisões em cascata refletidas nas inúmeras trajetórias de tratamento, de modo que esses métodos apresentem resultados que forneçam informações significativas de apoio às decisões nas políticas de saúde^19^.

O alto custo das tecnologias da MP pode agravar as desigualdades na saúde e pôr em risco a sustentabilidade de seus sistemas, especialmente em países de média e baixa renda. A avaliação dessas inovações tecnológicas exigirá não somente um novo conhecimento técnico, mas possivelmente também um outro processo nos órgãos de ATS, dado o ritmo de inovação e a regularidade de atualização de algoritmos e aplicativos^20^.

### Olhares sobre o Passado e o Presente para Refletir o Futuro da ATS

A formulação da PNGTS aconteceu entre 2004 e 2010, momento de ampliação das políticas de proteção social e de ações afirmativas. A institucionalização da ATS no SUS foi cristalizada com a criação da Conitec em 2011. Sua implementação se deu na conjuntura de 2013 a 2018, um período turbulento de regressão democrática, degradação institucional contínua e crise econômica, especialmente marcado por 2016, ano da ruptura democrática do impeachment da Presidente Dilma Rousseff e a extinção de todas as secretarias e ministérios ligados à ampliação de direitos ou a políticas distributivas^21^.

A instituição de um órgão nacional de ATS, como a Conitec, é uma tarefa difícil que exige muitos recursos e requer um período de amadurecimento e de compromisso político para se tornar efetiva e sustentável^22^. Desde 2016, a Conitec tem perdido sua força política e enfrentado estratégias de desestabilização, com substituições importantes no seu corpo técnico. Além disso, o ceticismo velado em relação às políticas fundamentadas em evidências científicas tornou-se um negacionismo científico explícito após a eleição de 2018^23^. Um exemplo claro dessa posição foi a decisão do governo atual de zerar a alíquota de importação do Zolgensma, o medicamento mais caro do mundo – cuja dose custa R$ 12 milhões – em julho de 2020, quando o medicamento não possuía registro na ANVISA.

Em resposta às perguntas iniciais, podemos dizer que há um equilíbrio instável entre o uso da ciência para informar a política e o uso político da ciência. A política de ATS tem procurado ser informada pela ciência, mas ainda existem lacunas técnico-científicas a serem preenchidas para que esse processo seja realmente fundamentado na evidência científica. Ela tem sido usada a serviço da política e atualmente está em risco por não ser valorizada no discurso político vigente.

A sobrevivência dessa política dependerá não só da ciência, ou seja, do fortalecimento da qualidade científica e técnica, mas sobretudo da arte de orquestrar os interesses dos sistemas político e judiciário, bem como da comunidade epistêmica e da sociedade civil, de forma a favorecer a inserção da ATS em uma política de saúde que fortaleça o SUS.

Portanto, aguardamos a reversão desse movimento pendular de contra democracia e defendemos que a nova ATS deixe de ser uma política de governo e se estabeleça como uma política de saúde e uma política social capaz de orientar os processos cada vez mais complexos de tomada de decisão no SUS. Esperamos que a ATS possa oferecer um processo transparente, legítimo e justo, contribuindo com o acesso às inovações tecnológicas de acordo com as necessidades da população e com o valor social dessas tecnologias, em respeito aos princípios da universalidade, da equidade e da integralidade. Dessa forma, padrões de segurança, qualidade e eficiência do SUS poderão ser garantidos e sua sustentabilidade, assegurada.

## Footnotes

a Refere-se aos resultados materiais concretos, à configuração de diretrizes, planos e programas políticos.

b Refere-se aos processos de negociação política, geralmente conflituosos, relacionados ao exercício do poder.

c Conceito operacionalizado por Peter Haas e Emmanuel Adler, refere-se a uma “rede de profissionais com uma reconhecida expertise e competência em um domínio específico e um conhecimento legitimado e relevante para a política dentro de um domínio ou área de problema”. O conceito não se restringe à comunidade científica; inclui também profissionais que aplicam a mesma metodologia e compartilham da mesma fé ou crença na verdade e na aplicabilidade de formas particulares de conhecimento ou verdades específicas. Assemelha-se à definição sociológica mais ampla de paradigma de Kuhn, que é “uma constelação de crenças, valores, técnicas compartilhadas por membros de uma dada comunidade”.
